# Clinical features and mortality outcomes of people transferred from prison to forensic mental health units: a nationwide 14-year retrospective cohort study

**DOI:** 10.1007/s00127-025-02893-5

**Published:** 2025-04-09

**Authors:** James A. Foulds, Ruth Cunningham, Toni L. Pitcher, Chris Frampton, Stuart A. Kinner, Ben Beaglehole

**Affiliations:** 1 Department of Psychological Medicine, University of Otago Christchurch, PO Box 4345, Christchurch, 8140 New Zealand; 2 Department of Public Health, University of Otago, Wellington, New Zealand; 3 Justice Health Group, Faculty of Health Sciences, Curtin University, Perth, WA Australia; 4 Justice Health Group, Centre for Adolescent Health, Murdoch Children’s Research Institute, Melbourne, VIC Australia; 5 Melbourne School of Population and Global Health, University of Melbourne, Melbourne, VIC Australia

**Keywords:** Prisoners, Mortality, Forensic psychiatry, Mental disorders, Involuntary transfer, Health services utilization

## Abstract

**Purpose:**

To describe a cohort of people transferred from prison to psychiatric hospital care and their mortality outcomes.

**Methods:**

Retrospective nationwide cohort of people (n = 1320) transferred from prison to a psychiatric hospital in New Zealand from 2009 to 2022. Follow up commenced at the first transfer and ended on 30 June 2023 or death if earlier. Ministry of Health records were used to describe the cohort and their service utilization profile. Records were linked to official mortality data, and mortality ratios were calculated using publicly available life tables.

**Results:**

The cohort was 85% male and 55% Māori, with a median age of 31.2 years. Most had a psychotic disorder (74%) or bipolar disorder (11%) and there were high levels of coexisting substance use disorder. Follow-up duration ranged from 2 months to 14.5 years (median 7.5 years) after the first transfer, of which 17% was in a psychiatric hospital. The age and sex-standardised mortality ratio for the cohort compared to the New Zealand population was 4.7 (95% CI 3.6–5.9). Among deaths with a known cause, 60% were from natural causes and 40% were from injuries including suicide.

**Conclusion:**

Despite extended periods of psychiatric hospitalization there was high mortality among people in the cohort. Investment in targeted prevention and coordinated, continuous healthcare is needed for people with a serious mental illness who experience incarceration.

## Introduction

It has long been known that prisons worldwide contain many people with serious mental illness [1], some of whom will require transfer to a hospital to have their needs met. In recent decades there has been increasing concern about the effectiveness of mental health services in New Zealand and the level of unmet need for mental health care. This led to a national mental health inquiry in 2018 [2]. Alongside these broader concerns about mental health services in New Zealand, we have previously reported on the inability of forensic mental health services to keep up with the needs of people in prison [3] and the resulting negative impact on human rights [4]. Similar issues have been observed in other countries including the United Kingdom and Ireland [5, 6]. The situation for forensic mental health services in New Zealand has been made more challenging by a steep and sustained increase in the proportion of people remanded to prison while their legal matters are dealt with, despite a recent reduction in the total prison population [7]. People on remand often have high health and social needs because of the stress and uncertainty of their legal matters and adjustment to prison. The high numbers of people in New Zealand imprisoned soon after a psychiatric inpatient admission [8] also points to increasing severity and complexity of mental health problems among those received into prison.

The New Zealand prison population climbed steeply in the 2010’s, reaching a peak of almost 11,000 (an incarceration rate of about 200 per 100,000 people) in 2018 across 18 prisons [7] before dropping steeply in response to government policy interventions [9]. The proportion of Māori in New Zealand prisons is currently 53%, compared to 17% in the general population, reflecting long term negative impacts of colonization [10]. In New Zealand, involuntary mental health treatment cannot be enforced in prison, therefore people needing it must be transferred to a psychiatric hospital. Most of those transferred have an acute psychotic disorder, often with coexisting personality disorder and/or substance use disorder. Almost all transfers are to a secure ward in one of five regional forensic facilities. There are about 220 inpatient beds across the country, most of which are medium and minimum secure. People admitted into forensic mental health care receive intensive multidisciplinary treatment, with some being returned to prison once stabilized and others receiving a purely hospital-based pathway. Those in the latter group mainly comprise people found unfit to stand trial or not criminally responsible on account of insanity after a serious violent offence.

There are two legal pathways for hospital transfer from prison. Over 90% of transfers from prison to hospital result from a prison-based clinician initiating the transfer under Sect. 45 (S45) of the Mental Health (Compulsory Assessment and Treatment) Act 1992 (MHA). Less commonly, transfer can occur after a Court order pursuant to the Criminal Procedure (Mentally Impaired Persons) Act 2003. Court-ordered transfers can occur for psychiatric reports; as disposition of defendants found unfit to stand trial or not criminally responsible on account of insanity; or pursuant to an order made at sentencing.

Most people in prison have high and complex health needs [11]. They have very high rates of mental illness and substance use disorder [12] and are at markedly increased risk of deaths after release from incarceration, from a range of preventable causes [13]. In New Zealand, recent data show a standardised mortality ratio (SMR) of 3.3 after release from prison, with this figure being even higher for women [14]. People using secondary mental health services in New Zealand also have increased mortality with an SMR around 2 [15]. Despite recognition of the burden of mental health problems among people in prison [12], evidence on interventions aimed at the mental health of people in prison and their long term outcomes is quite limited [16], though continuity of mental health and housing support after release are important [17]. Better longitudinal studies on the mental health of people who experience incarceration have been called for [18].

In this study we used New Zealand national administrative data to describe the demographic profile and clinical diagnoses of all people transferred from prison to psychiatric care under S45 over a 14-year period. Considering the elevated mortality among people released from prison [13] and those in contact with mental health services [15] we also aimed to measure mortality outcomes as a reliably-recorded and objective measure of health inequity for this population.

## Methods

### Ethical approval

The Ministry of Health gave written approval to use the data. Institutional ethical approval was given by the University of Otago (Approval reference H24/0127 UOHEC H). Indigenous consultation with the Ngāi Tahu Research Consultation Committee and the University of Otago Christchurch Māori Research Expertise Rōpū was also undertaken.

### Study design and inclusion criteria

We identified a retrospective cohort of all people in New Zealand who were subject to a S45 order from 1 January 2009 to 31 December 2022, using routinely collected data from the New Zealand Ministry of Health Programme for the Integration of Mental Health Data (PRIMHD). Cohort members were entered into the cohort at the date of their first S45 order on or after 1 January 2009. Those who had been placed under such an order prior to 1 January 2009 and were still under that order were excluded. This cohort comprises over 90% of people transferred from prisons to forensic mental health units in New Zealand, the remainder being those who were transferred under a Court order for assessment or treatment. The PRIMHD dataset contains national data on all inpatient and outpatient contacts with the public sector specialist mental health system. New Zealand has very few private inpatient psychiatry facilities (and none that can receive admissions under S45), so this dataset captures close to 100% of the inpatient mental health contacts for the cohort of interest. We also recorded cross-sectional numbers of people on remand and total prisoner numbers in New Zealand for a series of year-end (December 31 st) census dates for the years 2009–2022 using published data from the Department of Corrections [7].

### Variables: case mix

Date of birth, sex, and ethnicity were extracted from the PRIMHD database. We used a prioritised ethnicity approach consistent with Ministry of Health ethnicity data protocols, whereby individuals who report multiple ethnic affiliations were classified into a single ethnic group in the following order: Māori; Pacific; other non-European; and European [19]. To examine outcomes for the indigenous Māori population, a binary Māori/non-Māori variable was also created, where individuals were classified as Māori if their ethnicity was recorded as Māori in any of the Ministry of Health ethnicity fields during any episode of care in the follow-up period. ICD- 9 and ICD- 10 clinical diagnostic codes from all inpatient contacts were extracted. Cohort members attracted multiple diagnostic codes over the study period. Most people subject to a S45 order have a chronic psychotic disorder. Therefore, a primary psychiatric diagnosis was assigned by first identifying all individuals who had a diagnosis of a psychotic disorder (ICD- 9 codes 295–299 or ICD- 10 codes F20–F29) recorded during any of their hospital admissions between 2009 and 2022, including admissions before the date of entry into the cohort. We then identified a non-overlapping group with bipolar disorder during any admission (ICD- 9 codes 296, excluding codes related to unipolar depression and ICD- 10 codes F30x–F31x). The rest of the sample was then categorized according to the primary diagnostic code during their first S45 admission, within the following categories: primary substance use disorder; other diagnoses; or no diagnosis. A history of a substance use disorder (alcohol, cannabis or amphetamine use disorders) was recorded as a secondary diagnosis if any diagnostic code related to these substances was listed during any inpatient admission from 2009 to 2022.

### Variables: clinical outcomes

Individual-level service utilization variables were extracted from PRIMHD, including all mental health clinical contacts (inpatient bed nights, seclusion episodes and outpatient contacts) and the date and type of order for all MHA orders since 1999. All mental health inpatient bed nights after the first S45 order were collated, and they were categorized as forensic medium or maximum secure; forensic minimum secure; or general adult.

### Variables: mortality outcomes

We used data from the Ministry of Health Mortality Collection, which combines information about death registrations with information on causes of death. PRIMHD and Mortality Collection data were linked using the National Health Index (NHI) number and anonymised by the Ministry of Health. The primary outcome was survival from the time of the first S45 order being made. The date of death and cause of death for all individuals who died between 1 January 2009 and 30 June 2023 were identified. Causes of death were grouped into the following categories according to their ICD codes: injuries, including self-injury, suicide, accidents and assaults; and natural causes, divided by organ system according to ICD cause of death codes. The date and location (community, hospital or prison) of the last mental health contact prior to death was identified.

### Analysis

Analyses were conducted using SPSS version 28. The trend in the number of S45 orders per year was explored with linear regression. The case mix and clinical outcomes of the sample were tabulated. We then calculated crude mortality rates with 95% confidence intervals (95% CI) using the dates of all deaths, including those without a recorded cause of death. Age and sex-standardised mortality ratios were calculated for the whole cohort and for Māori in the cohort using life tables from Statistics New Zealand [20]. Age-standardised mortality ratios were also presented by sex. Mortality ratios were not calculated for non-Māori because Statistics New Zealand does not publish population mortality statistics for non-Māori in the general population. Models to explore age-standardised survival by sex, ethnicity (Māori versus non-Māori) and diagnostic groups (psychotic disorder and substance use disorder) were fitted using Cox regression.

## Results

**Fig. 1:**
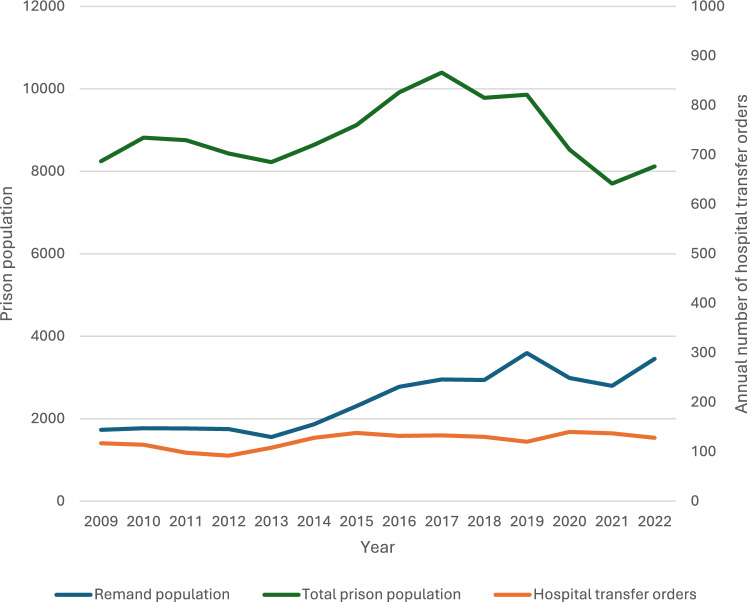
New Zealand remand and total prison population 2009 to 2022 and annual number of prison to hospital transfer orders^1^. 1. Linear trend in Sect. 45 hospital transfer orders: b = 2.4, p =.001. Total prison population and remand population are shown on the left- hand y axis, and Sect. 45 orders are shown on the right-hand axis

Between 1 January 2009 and 31 December 2022, there were 1715 new S45 hospital transfer orders made for 1320 people. Most of the cohort members (n = 1027, 78%) had no further S45 orders after their index hospital transfer. About one fifth (22.3%) of the cohort had at least one further order and the maximum number of orders was 9. The time from the date of the first S45 order to death or 30 June 2023 ranged from 2 months to 14.5 years (median 7.4 years), meaning cohort members were followed for a total of 9557 person years. As shown in Fig. 1, between 2009 and 2022 the number of S45 orders per year increased slightly (b = 2.4, p = 0.001), and it ranged from 92 to 140 per year.

### Case mix

Table 1 shows the demographic and clinical characteristics of the cohort and clinical outcomes. They were predominantly (84.6%) male, with a mean age at 33.7 years at entry into the cohort and a high proportion of Māori (55.4%). Table 2 shows the clinical characteristics of the sample. About three quarters (73.6%) had a primary diagnosis of a psychotic disorder, predominantly schizophrenia or schizoaffective disorder, and a further 11.2% had bipolar disorder. Almost half of the cohort had a recorded substance use disorder, predominantly alcohol, cannabis or amphetamine.

**Table 1:** Case mix and clinical outcomes for people transferred from prison to mental health care between 2009 and 2022 (n = 1320)

Measure	Number (%) or range (median)
*Demographics*	
Sex (percent male)	1118 (84.7)
Age at entry into cohort	17–84 (31.2)
Prioritised ethnicity:	
European	370 (28.0)
Māori	731 (55.4)
Pacific	152 (11.5)
Other	67 (5.1)
*Primary mental health diagnosis*	
Schizophrenia or schizoaffective disorder	812 (61.5)
Other psychotic disorders	160 (12.1)
Bipolar disorder	148 (11.2)
Alcohol or substance use disorder	39 (3.0)
Other^1^	65 (4.9)
No diagnosis or missing diagnosis^2^	90 (7.3)
*Substance use disorder diagnosis*^3^	
Any substance use disorder	585 (44.4)
Alcohol	422 (32.0)
Cannabis	276 (20.9)
Amphetamine	223 (16.9)
Opioid	39 (2.9)
*Legal characteristics*	
Number of prison to hospital transfers under S45 during follow up period	1–9 (1)
Mental Health Act Community Treatment Order any time before first Sect. 45 order	409 (31.0)
Mental Health Act Community Treatment Order within 12 months prior to first Sect. 45 order	127 (9.6)
Mental Health Act Community Treatment Order any time after Sect. 45 order	667 (50.5)
*Inpatient care variables*^4^	
Seclusion during any inpatient admission	875 (66.3)
Forensic medium or high secure inpatient bed nights	1–4817 (92)
Forensic minimum secure bed nights	1–3508 (308)
General adult psychiatric hospital bed nights	)

1. Includes patients whose primary diagnosis was a dementia or organic brain disorder; personality disorder; anxiety disorder; unipolar mood disorders; posttraumatic stress disorder; mental retardation; and other diagnoses

2. Patients for whom no primary diagnosis was recorded following a period of inpatient assessment

3. Recorded as a primary or secondary diagnosis at any time in the follow up period

4. Bed nights after the first prison to hospital transfer during the follow up period. The range and median number of bed nights for each facility type is shown for those who spent at least one night in that type of unit

**Table 2:** Mortality among people transferred from prison to hospital under Sect. 45 of the Mental Health Act on or after 1 January 2009 (n = 1320)

Group	Total N	Number of deaths	Person years of follow up	Crude mortality rate per 100,000 person years	Standardised mortality ratio^1^ (95% CI)
Whole cohort	1320	85	9557	842	4.7 (3.6–5.9)
Males	1118	71	8262	859	4.2 (3.2–5.4)
Females	201^2^	14	1292	1083	9.5 (5.0–16.2)
Māori	731	51	5285	965	2.7 (1.9–3.7)
Māori males	603	43	4449	966	2.4 (1.6–3.4)
Māori females	127^2^	8	829	965	5.5 (2.4–10.8)

1. Standardised mortality ratios (SMR) were calculated using population reference groups stratified by age, sex and ethnicity. For example, the SMR for Māori males uses Māori males in the general population as the reference group, with mortality bands in 5-year intervals

2. One person with unknown sex was excluded from sex-specific analyses

### Clinical outcomes

During follow-up, 93% of patients occupied at least one bed night in a medium or maximum secure facility. The number of medium or maximum secure bed nights occupied by cohort members ranged from 0 to 4817 nights (median 92). Those who had at least one night in a minimum secure unit (190; 14%) occupied from 1 to 3508 minimum secure bed night (median 308). Among those who had at least one night in a general adult psychiatric hospital bed (53%) bed nights ranged from 1 to 2349 (median 53). On average, cohort members occupied a forensic or general psychiatric hospital bed for 1.2 years during follow up, or 17% of the follow up period. About one third of cohort members (31%) spent time subject to a compulsory MHA order in the community at any time before their first S45 order, including 9.6% who were subject to a compulsory Community Treatment Order (CTO) in the 12 months prior to it, while half (50.5%) had a CTO at any time after their first S45 order. Two thirds (66.3%) experienced seclusion at any time in the study period.

### Mortality outcomes

During the follow-up period, 85 people died including 14 females. Table 2 shows the crude mortality rate and standardised mortality ratios for the cohort, and males, females and Māori in the cohort. This reports elevated mortality for the cohort, and for all subgroups within the cohort. Women had markedly elevated mortality, though the small number of deaths among women meant this estimate was imprecise. Cause of death information was available for 52 people while the remaining 33 were still awaiting the outcome of coronial investigation. Among those with cause of death information, 21 deaths were from injuries (including 6 by overdose/poisoning, 11 by suicide or self-injury, 2 by motor vehicle crash, 1 drowning, and 1 unspecified traumatic brain injury), and 31 were from medical causes (9 cancer, 15 cardiorespiratory and metabolic, 4 neurological, and 3 other). Relatively few deaths (n = 10, 12%) occurred within 12 months of the first Sect. 45 order. Among those who died, 64% had a contact with a mental health service clinician in the 30 days prior to death and 92% had at least one contact in the 12 months prior to death. Although the location of death was not recorded, the last mental health contact was in the community (including outpatient attendances and home visits) in 66/85 cases (78%). A further 12/85 (14%) had their last mental health contact in a psychiatric hospital or general hospital and 7/85 (8%) were in prison. Six of the seven patients whose last contact was in prison were seen by mental health services in the 12 months prior to their death, but only one was seen in the 30 days prior.

### Survival analysis

**Fig. 2:**
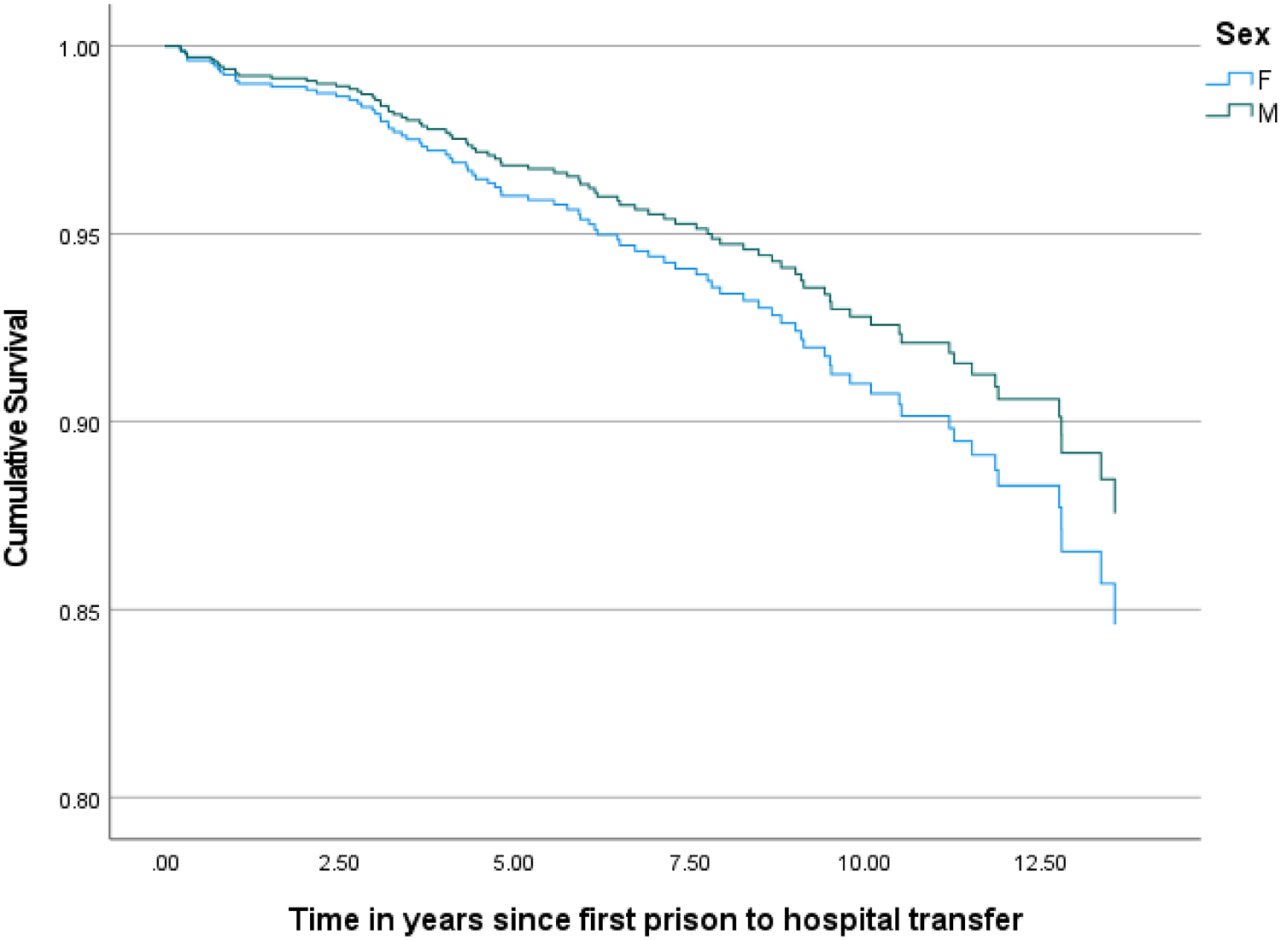
Age-adjusted survival stratified by sex: Cox regression model (p =.43 for effect of sex)

**Fig. 3:**
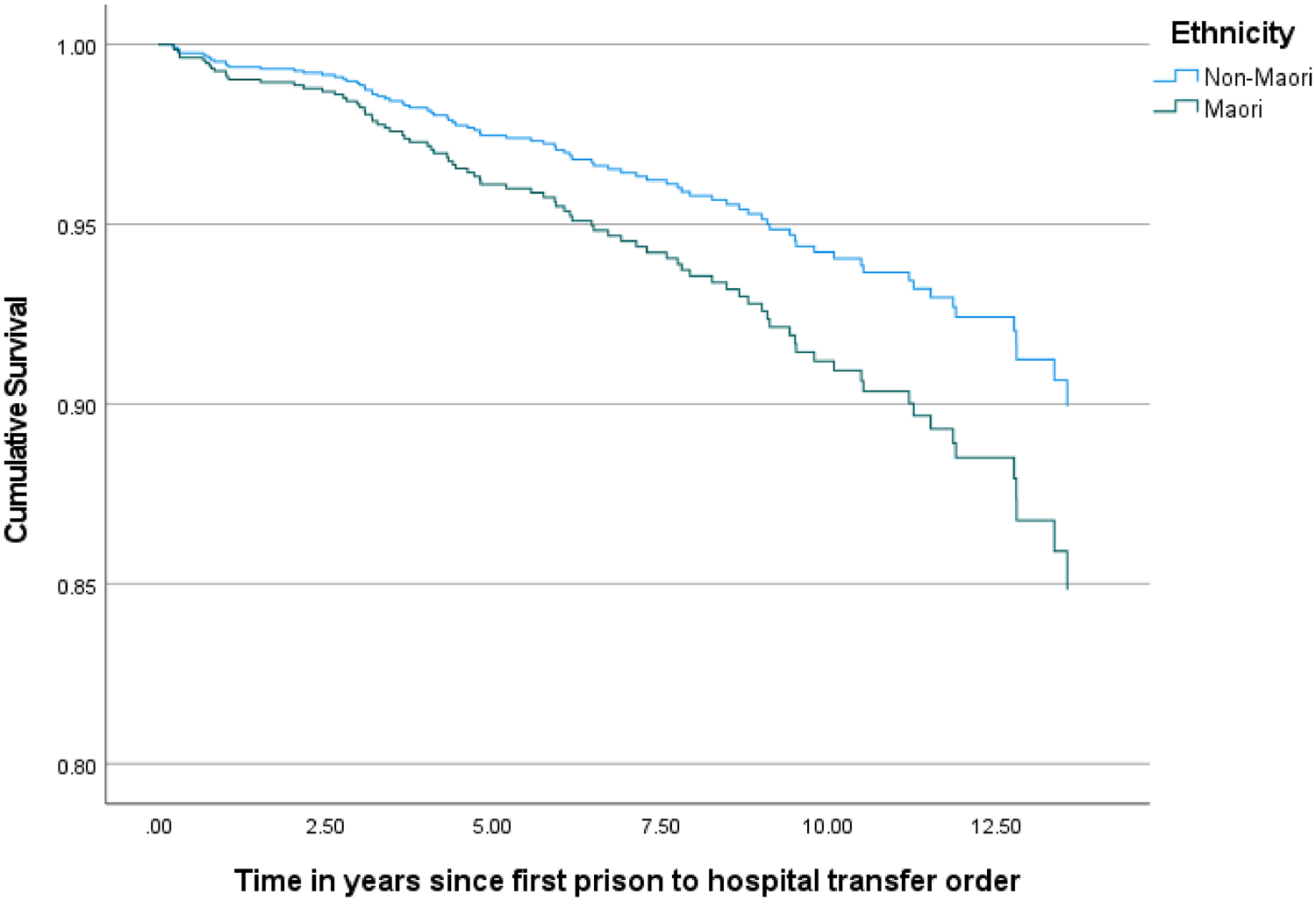
Age-adjusted survival stratified by ethnicity: Cox regression model (p =.05 for effect of ethnicity)

Figures 2 and 3 show the results of age-adjusted Cox regression models stratified by sex and ethnicity respectively. Sex was not associated with survival outcomes (hazard ratio = 0.79, 95% CI 0.45–1.4, p = 0.43; reference category = females) while non-Māori were less likely than Māori to die during the follow-up period (hazard ratio 0.64, 95% CI 0.41–1.00, p = 0.05). Coexisting substance use disorder (hazard ratio 1.19, 95% CI 0.80–1.83 compared to no substance use disorder) and the presence of a psychotic disorder (hazard ratio 1.15, 95% CI 0.74–1.80 compared to all other diagnostic groups) were not associated with survival.

## Discussion

There was a modest increase in the number of prison to hospital transfers between 2009 and 2022, though this number was likely constrained by hospital bed availability, and it was low relative to the total prison population. The cohort was predominantly male, with a median age in their early 30 s at the time of first transfer. Over 80% of people transferred from prison to psychiatric hospital units for treatment had a psychotic disorder or bipolar disorder. This suggests people in prison who may warrant intensive psychiatric treatment but are neither psychotic nor acutely behaviourally disturbed are seldom transferred to a psychiatric hospital in New Zealand. People who are unlikely to be transferred from prison to hospital care include those who are suicidal but not psychotic; people at risk of severe self-harm behaviour; and those with an acute crisis related to a personality disorder, adjustment disorder or posttraumatic stress disorder.

The high proportion of Māori (55%) in the cohort reflects their prevalence in the New Zealand prison population, at about 52% [7], compared to 17% in the New Zealand general population. Their true prevalence in our cohort may be even higher than we reported, as Māori are under-counted in health and disability data [21]. The over-representation of Māori in the cohort highlights health and social inequities and marginalization stemming from European colonization in New Zealand over more than two centuries [22]. Colonization has brought about widespread harms for Māori including loss of resources, disconnection from land and social systems, and intergenerational trauma. The 1840 Treaty of Waitangi was intended to provide constitutional protection for Māori, however the continuing inequities show successive New Zealand governments have failed to protect Māori and support their wellbeing and self-determination. Our data highlight a need for strategies to avoid criminalizing Māori with serious mental illness. The policy solutions must address the structural drivers of incarceration for Māori, which span multiple areas of government policy including health, education, justice and economic development. Better resourcing for Māori-focused health services is necessary to improve outcomes for Māori with serious mental illness who experience incarceration [23]. Such services need to be designed and delivered by Māori.

People in New Zealand prisons have access to a range of mental health treatment including medication and psychotherapy. These services are delivered by various providers including the Department of Corrections, non-governmental organisations, private providers, and forensic mental health services. However, the level of access to hospital care for people who are acutely or severely unwell is not equivalent to what is available in the community, with lengthy delays in transfer being reported [24]. Some of these patients are placed in special management units, often with solitary confinement, while awaiting transfer [25]. New Zealand’s regional forensic psychiatric services provide in-reach care to all New Zealand prisons but these services have not expanded in proportion to increasing demand [4]. This increase in demand is driven in part by a steep increase in people on remand while awaiting the outcome of their legal matters. The needs of this group are often higher than they are after sentencing, due to the stress of legal proceedings and dislocation from their usual living situation among other factors. The increasing prevalence of acute methamphetamine-related mental health problems, particularly psychosis, has also driven demand for forensic inpatient care. Over 10% of people in New Zealand prisons have a current (past 12 months) methamphetamine use disorder [12] and the prevalence of methamphetamine use disorder in our sample was 17%.

The challenges for forensic mental health services are not unique to New Zealand, and similar issues have been noted in the provision of prison mental health care in England and Wales [6] and Ireland [5]. While New Zealand has had prison mental health surveys that document the burden of mental illness across the whole prison population [12, 26], there is an urgent need for research into the level of unmet need for treatment, particularly acute mental health care that cannot be delivered in prison. As acutely mentally unwell people in prison are often managed in solitary confinement, research is also needed to estimate the extent to which human rights violations are taking place. Relatedly, there is a need for more transparent and robust processes to decide who is transferred from prison to hospital. Structured decision-making tools to guide admission to forensic units, such as DUNDRUM, can be a useful adjunct to existing decision-making processes [27]. However, these tools have not yet been implemented throughout New Zealand.

Once transferred out of prison, the cohort had a high level of ongoing mental health service utilization and healthcare need. They spent 17% of the follow up period in a psychiatric hospital bed, about 70% of which was in a forensic ward, and half received compulsory mental health treatment in the community. We found an over 40% prevalence of substance use disorder comorbidity in the cohort. However, this is almost certainly an underestimate, as it is far lower than the prevalence in the general prison population [12]. This could be due to diagnostic overshadowing, incomplete recording of substance use disorder diagnoses by clinicians, or incomplete diagnostic data in the PRIMHD dataset – likely it is a combination of these factors. More accurate data on substance use disorders in this population is vital, since substance use is an important modifiable risk factor for adverse outcomes including premature death, as it is for other groups experiencing incarceration [13].

The cohort had a more than fourfold elevation in age and sex-adjusted mortality compared to the general population. While the absolute number of deaths among females in the cohort was small, their risk was elevated ninefold compared to women in the general population. Māori, who made up over half the cohort, also experienced considerably elevated mortality compared to Māori in the general population. The relative increase in the risk of death found for this sample was greater than that found in a recent study of people released from incarceration in New Zealand, which found SMRs of 3.8 for women and 2.7 for men [14]. It was also higher than estimates of increased premature mortality among adults with psychotic disorders using mental health services in New Zealand, who have a standardised mortality rate about three times that of the general population [15].

Analysis of the available cause of death data showed that about 40% of the deaths were from injuries, predominantly overdoses and self-injury including suicide. Cancer and non-communicable cardiorespiratory diseases accounted for most of the non-injury deaths. Many cases were still under investigation by the Coroner, meaning the true ratio of injury to natural deaths is not clear. Recent data from the United States show an immense concentration of suicide deaths among people released from prison [28]. Considering the difficulty in predicting and preventing suicide at an individual level, there is a need for a broad and multisectoral approach that addresses the diverse needs of people released from incarceration [28].

The main strength of this study is that it is a national sample comprising all people transferred under the Mental Health Act from New Zealand prisons to psychiatric hospitals within the study period. The study excludes a small group of people transferred under different kinds of order, for example for Court-ordered inpatient psychiatric reports. The use of linked administrative data is both a limitation and a strength. Available administrative data in New Zealand provide limited detail on why people were transferred or their mental health status at the time. Diagnoses were from clinical coding data and as noted above, this is likely to under-report any disorders that are not accurately recorded in health records. Cause of death information was incomplete for about one third of deaths, chiefly because of delays in Coronial inquiries. Despite this, our use of national data spanning 15 years ensured near-complete ascertainment of mortality in the cohort. Relatively few members of the cohort are likely to have left New Zealand, and therefore to have missing outcome data, but we cannot exclude the possibility a small proportion of the cohort was lost to follow up. To the extent that this occurred, our estimates of mortality in the cohort would be conservative.

In summary, people who are transferred from New Zealand prisons to hospital for mental health treatment are predominantly young men with psychotic disorders or bipolar disorder, with an alarmingly high proportion of Māori, the indigenous people of New Zealand. They are at risk of premature death, which is likely to be driven by both illness factors and a range of social determinants including unstable housing and lack of employment. Such markers of social exclusion appear to be associated with risk of death in a dose–response fashion [29, 30]. The diverse nature of these risk factors implies a need for coordinated, multi-sectoral prevention strategies [13] and is not solely the role of forensic mental health and correctional services [28]. There also needs to be a commitment to providing high quality physical and mental health care for people in contact with the criminal justice system. Policy solutions aimed at this group of people must urgently be developed in partnership with Māori, who currently make up over half this population.

## Data Availability

Anonymised data were provided to the authors by the New Zealand Ministry of Health. The raw data are not publicly available and they cannot be shared without written consent of the Ministry of Health. Applications to access raw data should be directed to the primary author.
